# Approaching discussions about genetics with palliative patients and their families: a qualitative exploration with genetic health professionals

**DOI:** 10.1038/s41431-022-01179-7

**Published:** 2022-09-05

**Authors:** Stephanie White, Erin Turbitt, Jane L. Phillips, Chris Jacobs

**Affiliations:** 1 Graduate School of Health, University of Technology Sydney, Sydney, NSW Australia; 2 School of Nursing, Faculty of Health, Queensland University of Technology, Brisbane, QLD Australia

**Keywords:** Palliative care, Genetic testing, Genetic counselling, Ethics

## Abstract

Genetic information can provide clinical benefits to families of palliative patients. However, integration of genetics into mainstream medicine has not focused on palliative populations. We explored the views and experiences of genetic health professionals in addressing genetics with palliative patients, and their families. We conducted an interpretive descriptive qualitative study with genetic counsellors and clinical geneticists using interviews and focus groups. Findings were generated using reflexive thematic analysis. Three themes were identified: (1) Focusing on the benefit to the family, (2) The discomfort of addressing genetics near end-of-life and (3) “It’s always on the back-burner”: Challenges to getting genetics on the palliative care agenda. Participants discussed the familial benefit of genetics in palliative care alongside the challenges when patients are near end-of-life. They perceived genetics as low priority for palliative care due to misunderstandings related to the value of genetic information. Acknowledging the challenges in the palliative care context, genetic health professionals want improved service leadership and awareness of the familial benefits of palliative genetic testing. Strong leadership to support genetic health professionals in addressing these barriers is needed for the benefits of genetic information to be realised.

## Introduction

Identifying the genetic contribution of a palliative patient’s condition has utility for their family, as it offers the possibility to reduce morbidity and mortality if preventative measures are indicated and actioned [1]. However, up to a quarter of patients with life-limiting conditions, and their relatives, may be missing the opportunity for genetic counselling or testing prior to the terminal phase of their disease [2]. While genetic and genomic testing (‘genetic testing’) close to end-of-life is unlikely to benefit the patient, it could help family members assess their own genetic risk and make health, reproductive, social and financial decisions [3, 4]. Clinicians providing active treatment may not identify eligible patients for genetic testing early in their disease trajectory because of low genetics knowledge, competing clinical priorities or patients bypassing traditional treatment pathways [5]. Palliative care then becomes the final opportunity to conduct a genetic risk assessment and, if indicated, collect a DNA sample for the future benefit of family members.

Genetic counselling guidelines recommend adopting a person and family-centred approach, which suggests genetic health professionals should be conscious of the unique ethical and practical issues affecting palliative patients, and their families [6]. However, little is known about the views and experiences of the workforce providing genetic counselling and testing to palliative patients. First-person accounts and peripheral reports describe the emotional impact of genetic health professionals’ close proximity to death and grief [7, 8]. Further work is needed to understand the views and experiences of genetic health professionals when discussing genetic issues with palliative patients, and their families.

Alternatives to traditional clinical genetics pathways, including mainstreaming (non-genetic health professionals managing genetic testing) and translational genomic research, are changing the nature of genetic health professionals’ interactions with palliative patients and their families [9, 10]. Genetic health professionals generally support new delivery models because of the rapid integration of genomics into mainstream medicine and increasing workforce pressure [11, 12]. However, mainstreaming into palliative care appears to have received less attention, despite patients’ interest to discuss genetic testing to address existing concerns about their family members’ future disease risk [13, 14]. Some palliative care health professionals report having the capability to discuss genetics with their patients, others have varied opinions on the relevance of genetics to palliative care and concerns about causing harm, but most desire further education and support to improve their confidence [15, 16]. Understanding the views of genetic health professionals about delegating responsibility and providing support to palliative care health professionals will build evidence for an intervention designed to support the integration of genetics into the palliative care setting [17]. Therefore, we aimed to explore genetic health professionals’ views and experiences of integrating genetics and genomics into the care of people with palliative care needs, and their families, including their perceptions of the barriers and suggestions to support integration.

## Materials and methods

### Design

We used an interpretive descriptive qualitative study design with online focus groups and semi-structured interviews [18]. These findings are a sub-set of data from a broader qualitative study that additionally recruited palliative care nurses and doctors (reported elsewhere) [15]. The study protocol was pre-registered: https://osf.io/h4gt9/.

### Theoretical approach

Due to the limited evidence about genetics in palliative care, we selected an inductive approach to explore the boundaries of our participants’ views and to ensure our findings were grounded in the data [19]. Underpinned by a pragmatic epistemology, we aimed to generate findings relevant to the Australasian setting, but that could also inform stakeholders in comparable countries [17].

### Participants and sampling

We invited Australian and New Zealand genetic counsellors and clinical/cancer geneticists (including trainees) via their professional organisations (see supplementary file for further detail). Organisations sent an email blast or included the invitation in their newsletter. We published the invitation on our professional Twitter accounts and asked participants to snowball the invitation.

### Data instrument & collection

We developed a semi-structured focus group and interview guide informed by the World Health Organization Integrated Care for Chronic Conditions framework and existing literature [5, 20]. We piloted the guide with two genetic health professionals who suggested reordering two questions and clarifying that we wanted responses about malignant and non-malignant cohorts. We asked about experiences of genetic discussions with palliative patients and their families, barriers and facilitators, and perceptions of palliative care health professionals’ and organisations’ roles (e.g. hospitals or health services) in integrating genetics into the palliative care setting (interview guide in supplementary file).

Participants completed a demographic survey to provide context to their responses. We opted not to collect specific geographical location (e.g. state) or qualification/training status (e.g. clinical geneticist vs. clinical genetics fellow) to reduce the chance of participant identification. We conducted semi-structured interviews and focus groups via Zoom (except one in-person interview) due to COVID-19 limitations [21]. We prioritised focus groups to encourage fluidity of ideas and reduce social desirability bias, but offered one-on-one interviews if the individual preferred [22]. S.W conducted all individual interviews and moderated two focus groups and J.P moderated one focus group. Either S.W, C.J or J.P acted as an ‘observer’ at each focus group to take notes and to provide feedback and a summary to the moderator [23]. Interviews and focus groups were audio- and video-recorded on Zoom, transcribed verbatim and de-identified [21]. We returned transcripts to participants for member checking. We made a pragmatic decision to discontinue data collection when no new information related to the research questions was being identified in subsequent interviews. We acknowledge there is always potential for additional insights from continued data collection and agree with arguments that declarations of data saturation are incongruent with reflexive thematic analysis [24].

### Data analysis

Using inductive reflexive thematic analysis, we employed NVivo V12 to code transcripts and Microsoft Excel and Word to develop themes relevant to the research question [25, 26]. S.W led the analysis. C.J. co-coded two transcripts to engage with S.W. about data interpretation and resulting codes. We revised codes over several iterations before organising into initial themes. S.W. and E.T. met weekly over ten weeks to discuss and develop themes, with monthly input from the C.J. and J.P. We actively sought disconfirming cases.

### Reflexivity

As a clinical genetic counsellor, I (S.W.) considered myself an ‘insider’ to the participants [27]. The advantage of being an insider is easier access to participants, shared language and concepts, and a rich understanding of the topic with the potential to notice important subtleties. However, the disadvantage is the potential to introduce bias from pre-formed opinions and lack of objectivity. To mitigate these risks, I kept a reflexive journal, drew upon the collective qualitative research training of the study team and engaged in critical discussions about theme development to ensure our findings were true to our participants’ views.

### Ethics

We recorded verbal consent (verbal consent script in supplementary file). Participants with a pre-existing relationship with the interviewer were given additional reminders that non-participation would not harm their relationship with the research team. The University of Technology Sydney Human Research Ethics Committee granted ethics approval (ETH20-5046/20-5347).

## Results

We conducted three focus groups (two with four and one with five participants) and 13 one-on-one interviews between October and December 2020, totalling 26 genetic health professional participants. Focus groups lasted between 54 to 58 min (average 56 min) and interviews lasted between 21 to 52 min (average 29 min). There were ten (38.46%) medical doctors (clinical/cancer geneticists and clinical/cancer fellows/trainees) and 16 genetic counsellors (61.54%). Most were female (*n* = 21, 80.77%), worked in a metropolitan area (*n* = 21, 80.77%), public setting (*n* = 23, 88.46%) and had 0–5 years’ experience (*n* = 11, 42.31%) (Table 1). Two additional genetic counsellors expressed interest in participating; one was lost to follow-up and one did not participate due to scheduling issues.

**Table 1:** Participant demographics (*N* = 26).

Participant	Sex	Age range	Discipline	Years of experience	Work location^b,c,d^	Work sector
P1*	Female	31–45	Genetic counsellor	6–10	Regional	Public
P2*	Female	31–45	Genetic counsellor	6–10	Metropolitan	Public
P3^	Female	46–60	Genetic counsellor	>15	Metropolitan	Public
P4*	Male	31–45	Genetic counsellor	0–5	Metropolitan	Public
P5	Female	31–45	Genetic counsellor	6–10	Regional	Public
P6	Female	31–45	Genetic counsellor	0–5	Metropolitan	Public
P7*	Female	18–30	Genetic counsellor	0–5	Metropolitan	Public
P8*	Female	18–30	Genetic counsellor	0–5	Metropolitan	Public
P9^	Female	31–45	Genetic counsellor	0–5	Metropolitan	Public
P10^	Female	31–45	Genetic counsellor	11–15	Metropolitan	Public
P11	Female	46–60	Genetic counsellor	>15	Rural	Public
P12^	Female	18–30	Genetic counsellor	0–5	Regional	Public
P13	Female	31–45	Genetic counsellor	6–10	Metropolitan	Public
P14	Female	18–30	Genetic counsellor	0–5	Metropolitan	Public
P15	Female	31–45	Genetic counsellor	>15	Metropolitan	Public/private^e^
P16	Female	31–45	Genetic counsellor	11–15	Metropolitan	Public
P17~	Male	31–45	Medical doctor^a^	0–5	Metropolitan	Public
P18~	Female	31–45	Medical doctor	0–5	Metropolitan	Public
P19~	Male	>60	Medical doctor	>15	Metropolitan	Public
P20	Female	31–45	Medical doctor	0–5	Metropolitan	Public
P21~	Female	31–45	Medical doctor	0–5	Metropolitan	Public
P22	Female	>60	Medical doctor	>15	Regional	Public/private^e^
P23	Male	46–60	Medical doctor	>15	Metropolitan	Public
P24	Female	46–60	Medical doctor	11–15	Metropolitan	Public
P25	Male	46–60	Medical doctor	>15	Metropolitan	Private
P26	Female	>60	Medical doctor	>15	Metropolitan	Public

*Focus group one, ^Focus group two, ~Focus group three.

^a^All medical doctors were either clinical geneticists, cancer geneticists or clinical/cancer genetics fellows or trainees.

^b^Metropolitan: Within a major capital city (also known as ‘urban’).

^c^Regional: A city or town that lies outside of a major capital city.

^d^Rural: All areas that lie outside of metropolitan or regional areas.

^e^Equal mix of public & private work.

Participants had palliative experience predominantly in cancer genetics, and to a lesser extent in general clinical genetics, research and neonatal intensive care settings. Most interactions occurred in outpatient clinics. Some patients had genetic counselling during active treatment with supportive palliative care, while others were in their last days or weeks of life. For patients at end-of-life, DNA-banking, rather than genetic testing, was more common.

Three themes were identified: (1) Focusing on the benefit to the family, (2) The discomfort of addressing genetics near end-of-life and (3) “It’s always on the back-burner”: Challenges to getting genetics on the palliative care agenda. The third theme consists of two subthemes: (a) Burden of proof: Instilling the value of genetics in palliative care, and (b) “Individuals can only do so much”: Finding solutions in the absence of service leadership.

### Theme 1 – Focusing on the benefit to the family

Participants described the importance of the family unit when discussing genetics with palliative patients. They explained the main reason for testing was to elucidate relevant genetic information for relatives, rather than for the patient’s clinical benefit.

It’s a very different consult to our regular consults. It’s not so much about that patient, but the family, and a lot of the discussion is probably more so with the family (P1)

Their experience was that relatives often initiated referrals to the genetics service and were engaged in learning about their risk. Participants built relationships with these families, providing continuity of care before and after the patient’s death.

But the best thing about it is we will actually […] form relationships with not just that individual, […] you actually form a relationship with a family (P18)

Some participants described the legal and ethical challenge of family-centred care when health systems preference individual autonomy over familial benefits. They described cases where they could not discuss relevant genetic information with the family, because they did not have consent from the palliative patient.

An issue […] is when there’s issues with consent to share information with other relatives. So, just say a patient has died, we’ve got the contact for someone in the family [but] they’re not the engaged person, […] there’s a niece or someone more distantly related who is more engaged in the process. I think that’s a big issue (P2)

Participants were sensitive to families’ vulnerability in an end-of-life context, but most thought they were grateful for the opportunity to discuss the genetic implications of their relative’s disease. There was also a sense from participants that a family-centred approach was in-line with the palliative patient’s wishes.

There are often classic examples of needing to give people the chance to tell their story, […] they’re usually really grateful to have the opportunity to reflect on what’s going on and what it means for other people in the family (P16)

Participants had experience working with families who were unaware a DNA sample from their late relative would have helped evaluate their own genetic risk, which meant relatives could only be provided with empirical risk information rather than an individualised assessment. They noted the family’s frustration that genetics had not been discussed while their relative was alive.

Parents, who I saw […] for genetic counselling, where there wasn’t enough information were really quite cross with their doctors. That it hadn’t been presented to them in a way that they understood the information would be helpful to them for the future (P19)

Participants favoured addressing genetics with families while their relative was still alive so they did not miss the opportunity of obtaining a DNA sample for the family’s benefit.

So bringing it up […] obviously there’s a lot of distress going on, but I think it’s […] probably not more distressful than losing the opportunity and then the family not having had that opportunity (P10)

### Theme 2 – The discomfort of addressing genetics near end-of-life

Most participants thought palliative patients wanted to engage with genetics to leave a legacy, make meaning from their illness and for reassurance their family would have access to important information. However, they acknowledged the value of genetic information depended on patients’ and families’ personal values, which was difficult to assess when they were providing genetic counselling near end-of-life.

The other biggest challenge is […] you don’t have rapport and they don’t know who you are […] we’re just arriving at the very, very last minute […] you feel like an intruder in something that is such a private thing (P15)

Approaching family members to discuss genetics could be challenging because the conversation was taking place at an emotionally difficult time.

In these circumstances, you usually want someone who’s a close genetic relative, […] and then you’re talking to them at what’s usually a really awful time, initiating that conversation at that point is quite difficult (P7)

However, there was a sense among participants that it was important to recognise and overcome their own feelings of discomfort about having genetic discussions with palliative patients, and their families.

We all have to overcome our own discomfort in raising these issues with vulnerable people. And that takes quite a bit of doing (P19)

Participants found navigating discussions with palliative patients more difficult when important information was missing from their referral, such as prognosis, competency, family dynamics or circumstances.

One thing that I have found challenging is […] figuring out who’s the appropriate person [to contact] in the family. And sometimes that’s not really made clear on the referral (P7)

Participants remarked that palliative patients do not want to have a detailed discussion about genetic testing, even if they are willing to have a DNA sample collected.

Some people are just so unwell that they don’t want to […] have an appointment. They’re happy to have the test, but they just don’t want to go into everything (P13)

Discussions to obtain consent for genetic testing were managed by reducing or simplifying the information imparted. Participants wanted to convey the most important concepts, while not overburdening patients with irrelevant information. However, they described feeling conflicted about whether they were fulfilling their duty to obtain informed consent.

I’m not ever going to make someone listen to me […] if they’re not interested. But the thing that makes me uncomfortable is that even if I’m confident that they’re on board […] that they’re actually signing a piece of paper, which states that they understand things, which I really know that they don’t (P12)

### Theme 3 - “It’s always on the back-burner”: challenges to getting genetics on the palliative care agenda

#### Subtheme - Burden of proof: instilling the value of genetics in palliative care

Participants conveyed their sense that genetics is not a priority for palliative care health professionals because of misunderstandings related to the value of genetic information. Some speculated that late referrals to palliative care (for example, from oncology) might affect the palliative care health professionals’ ability to identify the need for a genetics discussion. Nonetheless, participants wished genetics were higher on their priority list so discussions could occur as early as possible in the patient’s disease trajectory.

So genetics, […] it never really has a priority. It’s always on the back burner, […] it’s not as [much a] quantifiable benefit as […] other areas of acute medical practice (P25)

Participants thought palliative care health professionals might avoid discussions about genetics because they believe another specialist has already addressed it, have concerns about harming patients or do not see genetics as relevant or part of their role.

You know, I’ve heard things said […] to families and patients, “Do you really want to spend your last days focusing on whether this might be hereditary or not, instead of just enjoying what time you have left?”, which is really disconcerting to hear, because I think both can be done (P24)

Noting palliative care health professionals’ expert communication skills, participants thought basic genetics education, particularly related to the importance of the proband sample and process of DNA banking, could be sufficient to prepare them for genetics discussions.

I think some of it comes from a misunderstanding that we actually need to test the person with the cancer diagnosis in the first place to get any useful information for the family (P1)

Participants felt responsible for providing education, but found it difficult to find time to deliver ongoing, concise and targeted education, due to the various cancer types and non-malignant conditions palliative care health professionals’ encounter.

I guess, it’s on us [to be] finding the channels to get in there, to let people know that we’re here […] There’s so many MDT meetings that we could be attending, but, you know, I can’t be everywhere at once (P5)

Participants described the value they could add to conversations about genetics with palliative patients and families. However, some wanted to improve their own palliative care knowledge to ensure they manage these discussions appropriately.

From a genetic counselling point of view, I would be keen to […] have had more training in the space. I think those, particularly end-of-life conversations, they’re quite confronting (P14)

#### Subtheme - “Individuals can only do so much”: finding solutions in the absence of service leadership

While a few participants described well-integrated services, most reported their services do not recognise the value of genetic information to palliative patients and families, with inadequate funding to develop solutions to existing barriers.

They come along and they say, “Yes, we want to help you, but there’s no money”. So, I think it’s that recognition that genetics […] is actually an integral part of all of these streams of medicine (P11)

Without clear leadership, participants noted that palliative patients (particularly those in private hospitals or from rural areas) were missing the opportunity to address genetics and wondered whether telehealth could help palliative patients overcome these inequities. Some described patients and family members overcoming access barriers by taking the initiative to seek out genetic testing for themselves.

I find that often when successful in the private setting, it’s because the family is motivated […] and very proactive in making sure the blood is collected […] So that’s often how it’s circumnavigated (P1)

Participants valued a multidisciplinary approach to care, but portrayed a lack of collaboration, communication and professional relationships between palliative care and genetic health professionals. They described feeling powerless as individuals in overcoming these barriers.

We’ve been in this building for three and a half years and I still have not worked out ways […] to get those buy-ins and having any kind of meaningful get together and ‘here’s what you are, here’s what we can offer’ and so on (P3)

Participants suggested several strategies to overcome barriers and support integration of genetics into palliative care (Table 2). These included workflow strategies, such as embedding a genetic counsellor within a palliative care team, tools to assess eligibility for genetic testing, such as a red flag checklist for new hospice admissions, and integrating genetic guidance into relevant policy.

**Table 2:** Strategies suggested by participants to support integration of genetics into palliative care.

SUGGESTED STRATEGY	SUPPORTING QUOTE
**Workflow strategies**	
Provide enough time and opportunity for patients and their families to consider whether genetic testing is right for them	I think it should [be] over multiple bites at the cherry. You know, just introduce the concept or explore the concept and then allow time to pass and answer questions as appropriate (P24)
Consider having a specialised or embedded genetic counsellor available for the palliative care service	I think that it’s quite important for genetic counsellors to have areas they specialise in, where professionals can call on them for advice. Because I think in a palliative care setting, you almost don’t need a physician because the diagnosis has been done (P20)
Encourage a palliative care health professional to champion genetics from the inside	You need […] somebody in palliative care who thinks it’s important […] and it’s not just got to be a doctor, it’s got to be the nurses. You really need somebody in nursing, who thinks it’s important (P26)
Encourage genetic and palliative care health professionals to attend the same multidisciplinary team (MDT) meetings	I think MDT meetings are the easiest way to integrate us in. Because I don’t think every department has the resources to have a genetic counsellor on staff, but the MDTs are an excellent opportunity to […] build the contacts to be able to have those discussions with each other (P5)
Liaise directly with palliative care health professionals who are involved in the patient’s care when a referral is received	Once I have spoken to the nurses or the physicians who are actually involved with that patient’s palliative care planning, they have been extremely helpful […] in terms of organising and carrying out a more satisfactory consultation for this family (P4)
**Strategies & tools to assess eligibility for genetic & genomic testing**	
Screen patients on admission to palliative care or hospice with a checklist, family history questionnaire, red flag document or digital application.	I would have thought some sort of triaged model with red flags, […] check around any questions about family risks, and maybe you’d even […] tailor it to the fact that people have children. That’s more likely to be at the front of their mind than if they don’t (P23)
Provide written material about genetics to patients and their families	What I would like to see is […] a sort of pack that both for […] doctors and for families around when family members are dying, that kind of almost raises some of those questions by default and then families can pick and choose (P17)
Ask patient and their family if they have any unmet need related to genetics	Maybe just checking with the patient […] “So have you been referred to genetics?”, “Has someone raised this with you that it could be hereditary?” [or] “OK, I can potentially be that liaison person, check in with genetics”. Because some people do forget that they’ve had anything through us (P13)
Consider reoffering the opportunity to palliative patients and families to discuss genetics	In that case […] we’d seen her previously and […] she either declined testing or hadn’t gotten around to having the blood taken and then realised the clock was ticking. And so desperately wanted to have the blood taken (P5)
**Service improvement strategies**	
Generate leadership by reflecting the value of genetics in relevant policy and/or guidelines	I think it would help if there was a national strategy on the integration of genomics into palliative care. […] I think it is quite important that you do have some sort of national leadership (P25)
Use telehealth services for patients receiving palliative care	One of the biggest barriers is that they’re too unwell, or that it’s just adding a burden to their appointments, so being able to stay at home […] in general I would say it’s probably been really positive for patients in general, but probably palliative care in particular (P13)
Improve capability of electronic medical records to share information between services	So how do they get access to medical records that sometimes might span over years? […] there’s a suggestion: electronic records that actually talk to each other. […] You can take a considerable amount of time to wade through health records to see if genetics has already been covered (P24)

## Discussion

We described three themes that illustrate the perceived clinical and psychological benefits of genetic information to families, the discomfort genetic health professionals can experience when providing genetic counselling near end-of-life, clinician and organisational level barriers preventing integration of genetics into palliative and potential strategies to overcome these.

Genetic health professionals emphasise the familial benefit, as opposed to the individual benefits, of genetic information in the palliative care context [28, 29]. The benefits described reflected broad definitions of utility, encompassing potential clinical and personal benefits (for example, the genetic test is of psychological value to the patient and family) [30, 31]. Discussion of the familial benefits overlapped with descriptions of family-centred care and relational approaches to autonomy in the palliative care literature, as a philosophy that centres the individual within their social system [32–35]. However, frameworks to operationalise family-centred approaches to care in Western health systems are often missing, putting health professionals in the difficult position of executing individualistic processes, despite knowing that families are integral to patient care [36]. The shared family-focused philosophies of palliative care and clinical genetics could be harnessed to design a family-centred intervention to support integration of genetics into palliative care.

Understanding a patient and family’s goals of genetic counselling or testing is key to building a trusting foundation to support shared decision-making [33]. Previous research describes deteriorating patient health, heightened emotions and limited time as barriers to discussing genetics, but our findings go further by suggesting these factors affect genetic health professionals’ ability to build relationships with and elicit the patient’s and family’s goals of genetic counselling and testing [37]. Families are often dealing with complex issues and making numerous decisions in the end-of-life stage, so despite the benefits of genetic information, health professionals can be uncomfortable broaching difficult discussions [38, 39]. Given the nature of interactions genetic health professionals have with grief and loss, additional training about palliative care may help to manage their discomfort [8]. Furthermore, we heard genetic health professionals adapt their approach to obtaining informed consent for genetic testing by reducing or simplifying information they convey to terminally ill patients. Patients at end-of-life may have decreased capacity to engage in complex discussions due to illness or delirium [40]. In addition, genetic health professionals may be considering and responding to the contextual ‘function’ of consent [41]. For palliative patients, the primary aim may be to establish any objection to genetic testing rather than focus on the individual clinical implications of the result [42]. To tailor appropriate approaches to genetic counselling, discussions about genetic testing and the associated medico-legal processes, further enquiries into patients’ and family members’ preferences for delivery, timing and content of discussions about genetics is urgently needed [43].

Akin to previous literature, our participants advocated for genetics to be introduced earlier in the patient’s disease course, rather than at end-of-life [44]. However, palliative care health professionals appear to be subject to well-known barriers to integration of genetics, such as low knowledge and confidence [15, 16, 45, 46]. Our participants echoed a general willingness to assist with improving palliative care health professionals’ genetics knowledge, but this was contingent upon time and resource constraints [47]. Our findings suggest genetic health professionals (in)ability to implement strategies to overcome structural barriers (for example, embedding a genetic counsellor within a palliative care team) was affected by a lack of funding and low awareness of genetic services at the organisational level [20]. Demonstrating the economic value of genetic testing for the benefit of relatives to organisations is complex [48]. While cascade testing rates are typically used to assess familial value, a more nuanced analysis combining health economics with ethical, legal and social implications may better illustrate the significance of genetic information, improve funding and support health professionals to implement strategies to support integration of genetics into palliative care [49].

### Strengths & limitations

This study combined a theory-informed instrument with an inductive approach to data analysis, allowing us to benefit from existing knowledge about genetic integration, while developing data-driven themes relevant to the palliative care context. A qualitative approach enabled exploration of participants’ views and experiences in this understudied area; however, generalisability is limited. Participants with strong views may have self-selected to participate, skewing the data with positive attitudes towards palliative-genetic integration, while negative or neutral views may not be represented. Most participants were female, working in public and metropolitan/urban settings. While this does represent a large portion of the genetic workforce in Australasia, views from diverse groups may not be captured here [50].

### Practice & research implications

Genetic health professionals and policy stakeholders can use these findings to increase awareness of the challenges genetic health professionals face when discussing genetics with palliative patients and families. Generalisability of our findings would improve if these themes were tested in a larger, quantitative study. Interventions to support integration of genetics into palliative care could harness the shared family-centred philosophies of clinical genetics and palliative care. Research with palliative patients and their families is required to understand their needs regarding genetic information.

## Conclusion

We identified three main themes that illustrate the centrality of the family when providing genetic counselling to palliative patients and their families, the discomfort of managing genetic issues near end-of-life, and highlight the practice barriers that are unlikely to be overcome without improved leadership to increase funding and implement targeted strategies. Cross-boundary collaboration between palliative care and genetics could focus on the shared value of family-centred care, while further research should elucidate the economic and personal value of genetic information to families to demonstrate the benefit of investing in the integration of genetics into palliative care.

## Supplementary information

Supplementary file

## Data Availability

Data pertaining to the development of themes for this study is available at Open Science Framework (https://osf.io/b2yqm/).
