# Supplementary material for: Approaching discussions about genetics with palliative patients and their families: a qualitative exploration with genetic health professionals
Source: Eur J Hum Genet. 2022 Sep 5;31(8):945–52. doi: 10.1038/s41431-022-01179-7 (PMC9441822; doi:10.1038/s41431-022-01179-7)
Supplement: Supplementary file 1 — Supplementary file [file 41431_2022_1179_MOESM1_ESM.docx]

Approaching discussions about genetics with people who have palliative care needs, and their families: A qualitative exploration with genetic health professionals

**Supplementary file**

Stephanie White^1^, Dr Erin Turbitt^1^, Professor Jane L. Phillips^2^ & Dr Chris Jacobs^1^

^1^ Graduate School of Health, University of Technology Sydney, NSW, Australia

^2^ School of Nursing, Faculty of Health, Queensland University of Technology, QLD, Australia

Table of Contents

[Appendix A: Recruitment strategy details 2](#_Toc103948873)

[Appendix B: Data collection instrument 3](#_Toc103948874)

[Appendix C: Verbal consent script template 5](#_Toc103948875)

# Appendix A: Recruitment strategy details

- Three professional bodies/special interest groups circulated an email invitation up to three times. These organisations were:
  - Human Genetics Society of Australasia (https://www.hgsa.org.au/)
  - Australasian Society of Genetic Counsellors (https://www.hgsa.org.au/asgc)
  - Australasian Association of Clinical Geneticists (https://www.hgsa.org.au/aacg)
- Two multidisciplinary research organisations circulated an email invitation to their members on two occasions:
  - Tranlsational Cancer Research Network (TCRN)
  - Maridulu Budyari Gumal (formerly known as SPHERE)
- A social media invitation was advertised through the research teams’ individual and associated professional Twitter accounts
- Participants were asked to forward the research invitation to any eligible and potentially interested genetics health professionals

# Appendix B: Data collection instrument

**Focus group/interview guide: Genetic health professionals**

**Welcome**

- Welcome each participant, introduce facilitator, observer and each other

**Overview**

- Thank everyone for their participation
- Explain purpose of focus group – to hear about views and experiences of integrating genetics into the care of people (and their families) with palliative needs, particularly to understand the barriers, facilitators and possible strategies to overcome these barriers. Describe how results will be used – to develop an evidence base about genetics in palliative care
- Explain why they have been invited and selected to participate – because they are a genetic counsellor or doctor working in genetics

**Consent questions**

- *Refer to Verbal Consent Script Template*

**Guidelines**

- If you haven’t already, please complete the demographics survey as soon as possible
- There are no right or wrong answers, only differing points of view. Even if you don’t agree with others, you must listen respectfully as others share their views
- Please respect each other’s privacy and confidentiality by refraining from speaking about the focus group outside of this forum
- As we are audio-recording, please ensure one person speaking at a time. Provide brief Zoom etiquette instructions
- We're on a first name basis
- If possible, please turn off mobile phones or turn to silent. If you must respond to a call, please do so as quietly as possible and rejoin us as soon as you can.
- My role as facilitator will be to guide the discussion among the group
- Thank everyone again. Ask each person to introduce themselves, their professional role and how long they have been working in their field

**Opening question (round-robin)**

1. Can you please tell me about your experience of genetics in the context of palliative care clients, or their families?

**Transitional questions**

1. In your experience, what have been some of the challenges of discussing genetics with palliative clients or their families? (*Prompts: What made this challenging? What were the successes? What made them successes?)*
2. What role do you think palliative care health professionals play in addressing genetics with palliative clients or their families? *(Prompts: Different roles for nurses/doctors/allied health? Any intervention more appropriate than others? Current role vs. future role? Could/should it be part of the role? Why/why not?)*
3. How do you think palliative clients and their families should be provided with genetic health information in an ideal world? (*Prompts: Timing, service, discipline)*
4. What is the role, if any, of larger organisations such as the hospital, professional palliative care organisations or government in helping palliative clients and families access genetic information?

**Key questions**

1. What are the main barriers and facilitators of discussing genetics with palliative clients and their families? (*Prompts: What are the benefits/harms to clients/families? Impact on workforce/workflow?)*
2. What are the facilitators/enablers?
3. If palliative care health professionals were asked by their service to start integrating genetics into their clinical practice, what would be the impact for you as a genetics clinician? (*Prompts: How would you feel? What would they need to make this happen? Educational needs? Impact on patients/families? Resource/staffing needs? Guidance?)*
4. Do you think it is appropriate and feasible for palliative care health professionals to integrate genetics with their clients and/or families?

**Ending question**

1. With all we have talked about today in mind, what is the most important point you’d like me to know about genetics in palliative care?
2. Is there anything you’d like add?

**Summary and closing**

1. *Facilitator/observer to summarise discussion.* Is this an accurate summary of what we have discussed?
2. *Facilitator to re-state the purpose of focus group discussion.* Is there anything we have missed? Any further thoughts anyone would like to add?

**Snowball/recruitment question**

1. Are there other individuals or groups who may be interested in participating in this research?
2. (If participant says yes) You’re under no obligation to do so, but if you’re willing, would you forward the research invitation to these individuals or groups? Alternatively, if you have permission to share their names and email addresses, I could forward them the research invitation myself.
3. (If participant says no) No problem, thank you for your time.

Thank everyone for coming. Ask if participant/s would like a summary of results once data analysis complete (explain email address will be retained).

*Please note: If the participant wishes to have a one-on-one interview instead, this schedule will be modified for that purpose.*

# Appendix C: Verbal consent script template

**VERBAL CONSENT TEMPLATE: UTS HREC REFERENCE NUMBER ETH20-5046/20-5347**

**Interviewee number:**

**Date:**

**Time:**

**Interviewer:**

*Key: Underlined areas indicate areas in which the script may differ, depending on whether consent the participant is consenting to a focus group or interview.*

“Thank you for agreeing to speak with me today about genetics in palliative care. The focus group/interview will take approximately 60 minutes. If at any point, you feel that you would rather not go on with the focus group/interview that is fine too”.

*[Wait for participant to confirm they are happy to continue, otherwise thank them for their time.]*

“Thank you. Now I just need to confirm some information about you, and I’m going to start recording. This will help us to accurately record the group discussion/your answers to the questions, but all this information will remain completely confidential. Is that OK?”

“First, I need to ask you some questions to confirm that you consent to participating. Remember, even after you've answered these questions, you can withdraw your consent at any time during the interview. However, it may not be possible to withdraw your data from the study results if these have already had your identifying details removed”.

The consent questions are: *(request response from each participant if in focus group):*

| **Question** | **Yes** | **No** |
| --- | --- | --- |
| Have you read the information contained in the participant information sheet? |  |  |
| Have you had an opportunity to ask questions and are you satisfied with the answers you have received? |  |  |
| Do you understand that there may be risks, such as the inconvenience of providing your time for this research and the potential to feel discomfort by the questions or your/others’ responses? |  |  |
| Do you understand that the research will produce reports, academic work or articles? |  |  |
| Do you freely agree to participate in this activity, with the understanding that you may withdraw at any time? |  |  |
| Do you agree to having this interview audio recorded and transcribed? |  |  |

(If answered NO to any of these – clarify and/or discontinue interview)

“If you have any concerns about the research you can contact myself, Stephanie White, or another member of the research team, Dr Chris Jacobs or Professor Jane Phillips.”

“If you would like to talk to someone who is not connected with the research, you may contact the Research Ethics Officer on 02 9514 9772 or Research.ethics@uts.edu.au and quote this number: UTS HREC Approval Number ETH20-5046.”

*Record if the participant declines to provide verbal consent:*

*Interview no. read the verbal consent script (or had it read to them) and agreed to participate on date: time: .*
